# Correction: Decoding distinctive features of plasma extracellular vesicles in amyotrophic lateral sclerosis

**DOI:** 10.1186/s13024-026-00955-z

**Published:** 2026-05-27

**Authors:** Laura Pasetto, Stefano Callegaro, Alessandro Corbelli, Fabio Fiordaliso, Deborah Ferrara, Laura Brunelli, Giovanna Sestito, Roberta Pastorelli, Elisa Bianchi, Marina Cretich, Marcella Chiari, Cristina Potrich, Cristina Moglia, Massimo Corbo, Gianni Sorarù, Christian Lunetta, Andrea Calvo, Adriano Chiò, Gabriele Mora, Maria Pennuto, Alessandro Quattrone, Francesco Rinaldi, Vito Giuseppe D’Agostino, Manuela Basso, Valentina Bonetto

**Affiliations:** 1 Istituto di Ricerche Farmacologiche Mario Negri IRCCS, Milan, Italy; 2 Department of Mathematics “Tullio Levi-Civita”, University of Padova, Padova, Italy; 3 Department of Cellular, Computational and Integrative Biology – CIBIO, University of Trento, Trento, Italy; 4 Consiglio Nazionale delle Ricerche, Istituto di Scienze e Tecnologie Chimiche “Giulio Natta” (SCITEC-CNR), Milan, Italy; 5 Centre for Materials and Microsystems, Fondazione Bruno Kessler, Trento, Italy; 6 Istituto di Biofisica, Consiglio Nazionale delle Ricerche, Trento, Italy; 7 ‘Rita Levi Montalcini’ Department of Neuroscience, Università degli Studi di Torino, Torino, Italy; 8 Department of Neurorehabilitation Sciences, Casa Cura Policlinico (CCP), Milan, Italy; 9 Department of Neuroscience, University of Padova, 35122 Padova, Italy; 10 NEuroMuscular Omnicentre (NEMO), Serena Onlus Foundation, Milan, Italy; 11 Department of Neurorehabilitation, ICS Maugeri IRCCS, Milan, Italy; 12 Department of Biomedical Sciences (DBS), University of Padova, 35131 Padova, Italy; 13 Veneto Institute of Molecular Medicine (VIMM), 35129 Padova, Italy

**Correction to: Molecular Neurodegeneration (2021) 16:52**

10.1186/s13024-021-00470-3

The original article omits a Data Availability statement which the authors note below:

“Anonymized patient raw data supporting the findings of this study are available from the corresponding authors upon reasonable request and after completion of a data-sharing agreement.”

